# Diagnosis and management of fracture-related infections in a low-income country: a prospective study comparing current practice to international consensus guidelines

**DOI:** 10.5194/jbji-11-53-2026

**Published:** 2026-01-29

**Authors:** Loïc Fonkoué, Elizabeth K. Tissingh, Cilia Ngang, Olivier Kennedy Muluem, Jasmine Sibatcheu Simo, Richard Douvamai, Jean Bahebeck, Olivier Cornu, Martin McNally

**Affiliations:** 1 Department of Orthopedics and Trauma, Yaoundé General Hospital, Yaoundé, Cameroon; 2 Department of Surgery and Specialties, University of Yaoundé I, Yaoundé, Cameroon; 3 Experimental and Clinical Research Institute, Université Catholique de Louvain, Brussels, Belgium; 4 Royal National Orthopedic Hospital NHS TRUST, London, UK; 5 King's Global Health Partnerships, School of Life Course and Population Sciences, King's College London, London, UK; 6 Department of Orthopedics and Trauma, Cliniques Universitaires Saint-Luc, Brussels, Belgium; 7 Bone Infection Unit, Nuffield Orthopaedic Centre, Oxford University Hospitals, Oxford, UK

## Abstract

Data on the implementation of international consensus guidelines for fracture-related infection (FRI) in low- and middle-income countries (LMICs) are scarce. This study assessed whether FRI diagnosis and management in an LMIC align with these recommendations. **Methods:** We conducted a prospective multicenter study across four tertiary hospitals in Yaoundé, Cameroon (September 2022–July 2025). All consecutive patients with a working FRI diagnosis were included. Confirmatory/suggestive diagnostic criteria and treatment strategies were assessed against consensus guidelines. **Results:** A total of 169 patients were included (mean age 39.4 ± 15.4 years; 72.7 % male). In 34.3 % of cases, FRI occurred without prior surgery, limiting applicability of the Willenegger and Roth classification. Clinical confirmatory criteria were present in 97 % of cases. Microbiological standards were seldom achieved: none fulfilled sampling quantity, and only 46.6 % met sampling method recommendations. A microbiological confirmatory criterion was documented in 36 patients (21.3 %); histopathology was rarely performed (1.2 %), and nuclear imaging was not used. Most patients (81.1 %) were on antibiotics before admission or surgery. The most common treatment strategies were suppressive antibiotic therapy (44.4 %); one-stage (11.2 %) or two-stage (10.7 %) debridement, antibiotics, and implant exchange (DAIEX); and debridement, antibiotics, and implant retention (DAIR; 9.5 %). Overall, 62.7 % of treatments deviated from consensus guidelines. **Conclusion:** Nearly two-thirds of FRIs in this LMIC setting were managed outside international consensus guidelines. While the consensus definition is applicable, diagnostic capacity remains limited and microbiological standards are often impractical. Context-adapted, evidence-based guidelines are urgently needed to improve outcomes where the burden is highest.

## 1 Introduction

Fracture-related infection (FRI) is highly prevalent in low- and middle-income countries (LMICs), particularly in sub-Saharan Africa, where its incidence can reach up to 52 % following open fractures (Fonkoue et al., 2023b; Kouassi et al., 2019; Whiting et al., 2019) and 5 %–9 % after surgery of closed fractures (Fonkoué et al., 2024b; Saris et al., 2006). Although it receives limited global attention, most FRI cases occur and are managed in LMICs (Tissingh et al., 2022). Various constraints, such as delayed access to care, limited diagnostic resources, and inadequate infrastructure, render their management particularly challenging (Tissingh et al., 2022; Metsemakers et al., 2023). As a result, LMICs are believed to bear the highest burden of FRI worldwide (Metsemakers et al., 2023; Tissingh et al., 2022).

International expert groups – including the Fracture-Related Infection (FRI) Consensus Group and the International Consensus Meeting (ICM) Orthopaedic Trauma Work Group – have issued standardized definitions, diagnostic criteria, and treatment guidelines (Govaert et al., 2020; Metsemakers et al., 2018; Obremskey et al., 2020). These guidelines, primarily developed by experts from high-income countries (HICs), rely on several key elements (Metsemakers et al., 2020; Rupp et al., 2023; Bezstarosti et al., 2019; Depypere et al., 2020a; Prada et al., 2022; Vicenti et al., 2024; Foster et al., 2020): a multidisciplinary team (MDT) approach, host optimization, surgery, and combined systemic and local antimicrobial therapy. The main surgical concepts are (1) debridement, antibiotics, and implant retention (DAIR) in the case of early infection with a good fracture reduction and stable construct and (2) debridement, antibiotics, and implant exchange (DAIEX), performed in one or multiple stages, or implant removal if the fracture has consolidated (Metsemakers et al., 2020; Marais et al., 2024a).

In addition to surgery, the second cornerstone of FRI treatment is antimicrobial therapy. Antibiotics should be withheld until deep tissue sampling, except in sepsis. Guidelines for microbiological analysis recommend five deep tissue samples and collaboration with infectious disease specialists for antimicrobial stewardship (Dudareva et al., 2021). Local antibiotics delivered via carriers are strongly encouraged (Metsemakers et al., 2020; Unsworth et al., 2024; Sliepen et al., 2022). Suppressive therapy is reserved for difficult-to-treat cases or when surgery is not feasible, with antibiotics continued until fracture union (Metsemakers et al., 2020; Tsang et al., 2024).

These recommendations have demonstrated improved outcomes in HICs (Rupp et al., 2023; McNally et al., 2022). However, their implementation in LMICs is hampered by financial barriers, lack of insurance, scarce diagnostic tools, uneven surgical expertise, and limited access to updated literature (Tissingh et al., 2022; Tsang et al., 2024; Metsemakers et al., 2023). Moreover, guidelines were developed without LMIC representation, raising concerns about applicability (Tissingh et al., 2022). To date, there is a critical lack of data on how FRI is diagnosed and managed in LMICs (Metsemakers et al., 2023; Tissingh et al., 2022; Tsang et al., 2024), although a context-adapted guideline has recently become available (ABJIN, 2025). Therefore, this study assessed diagnostic and treatment practices for FRI across tertiary hospitals in Cameroon, evaluated adherence to international guidelines, and identified areas for intervention to improve care in resource-limited contexts.

## 2 Patients and methods

### 2.1 Ethics approval

The Institutional Review Board of the University of Yaoundé I, Cameroon, approved the study protocol (Ethical clearance No 0953/UY1/FMSB). Patients or their legal guardians provided informed consent for participation.

### 2.2 Study design

We conducted a prospective, consecutive, observational cohort study including all patients diagnosed and treated for FRI in four tertiary hospitals in Yaoundé, Cameroon, between September 2022 and July 2025. All patients, of any age, in whom limb FRI was suspected or confirmed and documented as such in the medical records by the treating clinical team, were included. For each patient with a working diagnosis of FRI, investigators assessed the presence of confirmatory and suggestive diagnostic criteria according to the 2018 International FRI Consensus Group Definition (Metsemakers et al., 2018; Govaert et al., 2020).

### 2.3 Data collection

Data included demographics, comorbidities, index fracture details, clinical presentation, and laboratory and radiologic findings. All confirmatory and suggestive criteria were assessed, and FRIs were classified according to Willenegger and Roth (Willenegger and Roth, 1986). For each case, management was evaluated across multiple dimensions. An MDT approach was considered present if the case was discussed in a meeting with ≥2 specialists (orthopedic surgeon, plastic surgeon, microbiologist, infectious disease specialist, endocrinologist, or radiologist). Patients were “optimized” if comorbidities (e.g., diabetes, obesity, malnutrition, smoking, vascular disease, HIV) had been addressed pre-operatively.

Surgical data included delay from injury to FRI diagnosis, procedure type (DAIR, DAIEX, or other), number of surgeries, dead space management, local antibiotics, fixation, and soft tissue coverage. Microbiological assessment documented sampling method, number of samples, and pathogens. Antimicrobial therapy was evaluated for timing, duration, and adequacy.

Two investigators assessed adherence to core consensus principles using the algorithm by Depypere et al. (2020b). DAIR was appropriate if the implant was stable, symptoms were <10 weeks, host status was favorable, soft tissue was viable with sufficient coverage, and there was no intramedullary nail (Baertl et al., 2024; Vicenti et al., 2024). Suppressive therapy was deemed appropriate for type C hosts (Cierny and Mader) or during active fracture healing with callus formation (Tsang et al., 2024; Depypere et al., 2020b; Metsemakers et al., 2020). Debridement with implant removal was considered suitable after fracture union. In all other scenarios, implant exchange (DAIEX) in one or two stages was considered the appropriate strategy. Antibiotic therapy was considered appropriate if culture-specific antibiotics were administered for a total of 6 weeks in cases without an implant or following implant removal and for 12 weeks in cases of implant retention or exchange (Depypere et al., 2020a, b, Asim et al., 2024).

### 2.4 Statistical analysis

Statistical analyses were performed using SPSS software, version 26.0 (IBM Corp., Chicago, IL, USA). Categorical variables were expressed as frequencies and percentages, and continuous variables were expressed as means ± standard deviation (SD). Associations between timing of infection onset and treatment approach, along with adherence to guidelines and clinical outcome, were tested with chi-squared analysis. Statistical significance was set at p<0.05 .

## 3 Results

### 3.1 Patient demographics and index fracture details

**Figure 1:**
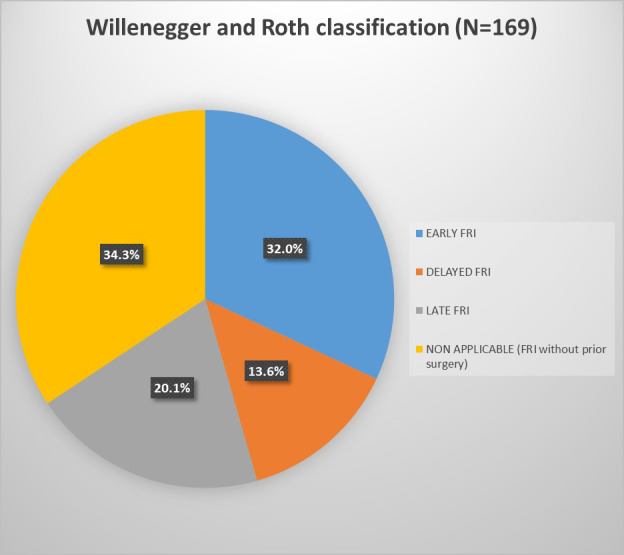
Division of cases according to Willenegger and Roth classification.

During the study period, 169 patients were diagnosed and treated for FRI. Baseline characteristics are summarized in Table 1. Most patients were young males from low–middle socioeconomic backgrounds and otherwise healthy (ASA I). FRIs mainly followed open fractures (63 %), involving predominantly the tibia and femur. Index fractures were most often fixed with external fixation or plates, but over one-third were managed non-surgically. The mean interval from injury to FRI diagnosis was 50.0 ± 106.7 d. According to Willenegger and Roth, FRIs were early in 54 cases (32.0 %), delayed in 23 cases (13.6 %), and late in 34 cases (20.1 %) (Fig. 1). The 58 patients without prior surgery – 51 open fractures and 7 treated by bonesetters – could not be classified with this system, as it is based on the interval between index surgery and onset of infection. This could not be applied as no surgical time point was available.

**Table 1:** Patient characteristics.

Variable	Value ( N=169 )
Age in years (mean ± SD, range)	39.4 ± 15.4 (5–80)
Gender: number of men (%)	130 (76.9 %)
Body mass index (kg m^−2^) (mean ± SD, range)	25.7 ± 5.1 (16.6–39.3)
Socio-economic level	
Poor ( < USD 60 per month)	13 (7.7 %)
Low (USD 60–250 per month)	80 (47.4 %)
Middle low (USD 250–500 per month)	57 (33.7 %)
Middle high (USD 500–1000 per month)	15 (8.8 %)
High ( > USD 1000 per month)	4 (2.4 %)
ASA classification^1^, number (%)	
1	112 (66.2 %)
2	32 (19.0 %)
3	22 (13.0 %)
4	3 (1.8 %)
Smoking, number (%)	14 (8.3 %)
Diabetes, number (%)	9 (5,3 %)
HIV, number (%)	12 (7,1 %)
Fracture localization	
Tibia and/or fibula	88 (52.0 %)
Femur	44 (26.0 %)
Foot	13 (7.7 %)
Humerus	10 (5.9 %)
Pelvic ring or acetabulum	6 (3.6 %)
Patella	4 (2.4 %)
Radius and/or ulna	3 (1.8 %)
Hand	1 (0.6 %)
Open fracture, number (%)	107 (63.3 %)
GA I	7 (6.5 %)
GA II	37 (34.6 %)
GA IIIA	33 (30.8 %)
GA IIIB	26 (24.3 %)
GA IIIC	4 (3.8 %)
Type of fracture fixation at index operation	
External fixator as definite treatment	47 (27.8 %)
Plate and screws osteosynthesis	40 (23.6 %)
Intramedullary nailing	14 (8.3 %)
Pinning/cerclage 6	5 (3.0 %)
Screw osteosynthesis	5 (3.0 %)
No previous surgery	58 (34.3 %)
Time from injury until onset of symptoms (days)	50.0 ± 106.7

SD = standard deviation; USD = US dollars; ASA = American Society of Anesthesiologists; GA = Gustilo–Anderson.

### 3.2 Diagnostic criteria

The prevalence of confirmatory and suggestive diagnostic criteria is shown in Table 2. All patients had at least one confirmatory criterion, with 164 (97 %) displaying a clinical confirmatory sign. Microbiological analysis was performed in 131 (77.5 %) cases, with confirmatory criteria met in only 36 (21.3 %) patients, largely due to insufficient sampling ( ≥2 samples in only 39.0 % of patients). Histopathology was done in only 2 cases (1.2 %), both positive. Among suggestive criteria, local signs of inflammation ( N = 65, 38.5 %), fever ( N = 56, 33.1 %), and wound drainage ( N = 51, 30.4 %) were most common. Elevated white blood cell (WBC) count and CRP were observed in 51 (30.2 %) and 105 (62.1 %) patients, respectively. Plain radiographic signs were present in 55 patients (32.5 %). Only 5 patients (3.0 %) underwent CT scans. No MRI, ^18^FDG-PET, or scintigraphy was performed. Pathogens were isolated from a single culture sample in 69 cases (40.8 %).

**Table 2:** Diagnostic criteria.

Variable	Criterion assessed N (%)	Criterion present N (%)
Confirmatory criteria		
Any confirmatory criterion	169 (100 %)	169 (100 %)
Confirmatory clinical criteria		
Fistula, sinus, or wound breakdown	169 (100 %)	117 (69.2 %)
Purulent drainage or pus	169 (100 %)	132 (78.1 %)
Confirmatory microbiological criterion (at least 2 cultures taken in only 51 cases)		
Phenotypically indistinguishable microorganisms isolated from at least two separate deep tissue cultures	131 (77.5 %)	36 (21.3 %)
Confirmatory histopathological criterion ( n=2 histopathological analyses)		
Histological presence of ≥5 PMNs/HPF	2 (1.2 %)	2 (1.2 %)
Histological presence of microorganisms	0 (0 %)	0 (0 %)
Suggestive criteria		
Clinical suggestive criteria		
Fever	169 (100 %)	56 (33,1 %)
Local clinical signs of inflammation	169 (100 %)	65 (38.5 %)
New onset joint effusion	169 (100 %)	24 (14.2 %)
Persistent, increasing, or new onset wound drainage	169 (100 %)	51 (30.4 %)
Radiological suggestive criteria		
Conventional radiograph signs	169 (100 %)	55 (32.5 %)
CT scan signs	5 (3.0 %)	5 (3.0 %)
MRI, FDG-PET, or WBC scintigraphy	0 (0 %)	0 (0 %)
Diagnostic biomarkers		
Elevated WBC count	169 (100 %)	51 (30.2 %)
Elevated CRP	169 (100 %)	105 (62.1 %)
Elevated ESR	169 (100 %)	100 (59.2 %)
Microbiological signs		
Pathogenic organism identified by culture from a single deep tissue or implant specimen	131 (77.5 %)	69 (40.8 %)

### 3.3 Microbiological findings

Of 131 patients (77.5 %) who underwent microbiological analysis, intraoperative biopsies were obtained in 61 (46.6 %) cases. The mean number of samples was 1.4 ± 0.5 (range 1–3); none had ≥5 as recommended. Cultures were positive in 105 patients (80.2 %). Infections were polymicrobial in 58 cases (44.3 %), and Gram-negative bacteria were predominant ( N = 97, 63 %).

**Table 3:** Microbiological characteristics.

	Frequency (%)
Variable	( N = 131)
Microbiological analysis	131 (77.5 %)
Sampling method	
Swab	35 (26.7 %)
Pus aspiration	35 (26.7 %)
Intraoperative deep biopsy	61 (46.6 %)
Number of samples per patient	
1	80 (61.1 %)
2	48 (36.6 %)
3	3 (2.3 %)
> 3	0 (0 %)
Positive culture	105 (80.2 %)
Single positive culture	69 (52.7 %)
Two positive culture with same microorganism	36 (27.5 %)
Polymicrobial infection	58 (44.3 %)
Total number of microorganisms	154
Gram-negative bacteria	97 (63.0 %)
Microorganisms isolated	
*Staphylococcus aureus*	31 (20.1 %)
*Klebsiella pneumoniae*	21 (13.6 %)
*Enterobacter cloacae*	18 (11.7 %)
*Escherichia coli*	14 (9.1 %)
*Pseudomonas aeruginosa*	9 (5.8 %)
*Proteus mirabilis*	8 (5.2 %)
*Enterococcus faecalis*	7 (4.5 %)
*Morganella morganii*	6 (3.9 %)
*Citrobacter koseri*	6 (3.9 %)
Group B *Streptococcus*	5 (3.2 %)
Coagulase-negative *Staphylococcus*	4 (2.6 %)
*Peptoniphilus asaccharolyticus*	4 (2.6 %)
*Acinetobacter baumannii*	3 (2.0 %)
*Providencia stuartii*	3 (2.0 %)
*Citrobacter freundii*	3 (2.0 %)
*Citrobacter sedlakii*	2 (1.3 %)
Others (*Enterococcus*)	6 (3.9 %)
Others (*Enterobacteriaceae*)	4 (2.6 %)

### 3.4 Management

**Figure 2:**
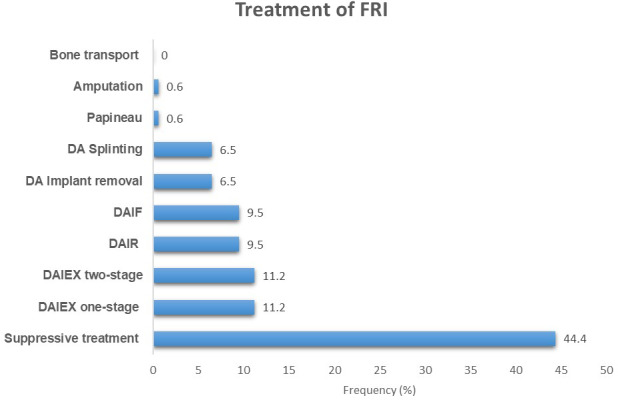
Distribution of cases according to the treatment performed. DAIR: debridement, antibiotics, and implant retention; DAIEX: debridement, antibiotics, and implant exchange; DAIF: debridement, antibiotics, and initial fixation in previously non-stabilized fractures; DA: debridement, antibiotics.

No MDT approach was applied. Suppressive antibiotics were the main treatment ( N = 75, 44.4 %). First intention treatment of FRI involved surgery in only 94 (55.6 %) cases (Table 4). The most commonly used procedures included one-stage implant exchange (DAIEX, 11.2 %), two-stage DAIEX with a cement spacer (Masquelet technique, 10.7 %), debridement and initial fixation in previously non-stabilized fractures (DAIF, 9.5 %), and debridement with implant retention (DAIR, 9.5 %) (Fig. 2).

Bone void fillers included bone cement and grafts (10.7 %). No ceramic-based carriers were employed. Local antibiotics were delivered as powder without carrier (24.8 %), antibiotic-loaded cement (10.7 %), and antibiotic-impregnated bone graft (grafts harvested and mixed with antibiotics for 1 h before implantation in the same surgical procedure) (7.1 %). In implant exchange cases ( N = 38), external fixation was the predominant stabilization method ( N = 36, 94.7 %), including monoplanar (63.8 %) and biplanar (36.2 %). No circular frames were used. Soft tissue reconstruction used local or rotational flaps in 19 patients (11.3 %), with no free flaps (Table 4).

**Table 4:** Management of FRI.

	Frequency (%)
Variable	( N = 169)
First intention treatment performed	
Surgical treatment	94 (55.6 %)
Non-operative treatment	75 (44.4 %)
Surgical treatment option performed	
DAIR	16 (9.5 %)
DAIEX single stage	19 (11.2 %)
DAIEX two-stage with cement spacer (Masquelet technique)	18 (10.7 %)
DAIEX two-stage without spacer	1 (0.6 %)
DAIF (debridement, antibiotics, and initial fixation)	16 (9.5 %)
Debridement, antibiotics, and implant removal	11 (6.5 %)
Debridement, antibiotics, and splinting	11 (6.5 %)
Papineau technique	1 (0.6 %)
Bone transport	0 (0 %)
Amputation	1 (0.6 %)
Local antibiotics	
Antibiotic powder	42 (24.8 %)
Cement with antibiotics	18 (10.7 %)
Antibiotic local injection	1 (0.6 %)
Antibiotic-impregnated bone graft	12 (7.1 %)
Ceramics with antibiotics	0 (0 %)
Soft tissue coverage	
Primary sutures	50 (29.5 %)
Secondary wound healing ± skin graft	25 (14.7 %)
Local fascio-cutaneous flap	13 (7.7 %)
Rotational muscular flap	6 (3.6 %)
Free flap	0 (0 %)
Systemic antibiotic therapy	
Mean total duration (in weeks)	11.1 ± 16.6
Appropriate antibiotic treatment	
No implant (6 weeks)	13 (56.1 %)
Implant retention or exchange (12 weeks)	20 (28.1 %)

DAIR: debridement, antibiotics, and implant retention; DAIEX: debridement, antibiotics, and implant exchange; DAIF: debridement, antibiotics, and initial fixation).

Prior to admission or surgery, 137 patients (81.1 %) were already on antibiotics. Mean antimicrobial duration was 11.1 ± 16.6 weeks. Excluding patients on suppressive therapy from this specific analysis, an appropriate antibiotic regimen was achieved in 13 patients (56.5 %) without an implant and in 20 patients (28.1 %) in whom an implant was retained or exchanged.

**Figure 3:**
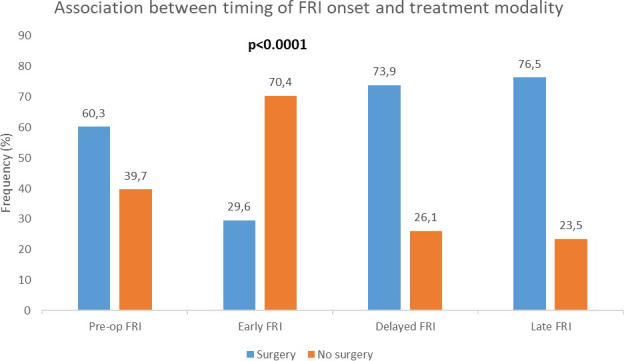
Association between timing of onset of FRI and treatment modality. There was a statistically significant association between the timing of FRI onset and the treatment (surgical or non-surgical) ( p<0.0001 ). Pre-op FRI: fracture-related infection diagnosed in the absence of previous surgical treatment.

There was a significant association between the timing of FRI onset and treatment modality (surgical vs. non-surgical) (Fig. 3). Surgery was most frequent in delayed (73.9 %) and late FRI (76.5 %) than in early FRI (29.6 %). Notably, 60.3 % of patients who developed FRI without prior surgery underwent surgical treatment ( p<0.0001 ).

### 3.5 Compliance with consensus guidelines

Treatment deviated from guidelines in 106 patients (62.7 %). Main reasons were inappropriate suppressive therapy (55.6 %), surgical indication not aligned with recommendations (29.2 %), or financial barriers preventing indicated treatment (15.2 %).

## 4 Discussion

Since the publication of the international FRI consensus guidelines for the diagnosis and management of FRI, developed by experts from HICs, there has been limited evidence from LMICs addressing this issue. Consequently, the applicability and implementation of these recommendations in resource-limited settings remain uncertain. This study addresses this knowledge gap by providing updated, context-specific data on the diagnosis and management of FRIs in LMICs, thereby helping to answer a critical question: how applicable are the current international guidelines in these settings (Tissingh et al., 2022)? 

### 4.1 Diagnostic criteria

FRI diagnosis relied on clinical confirmatory criteria in 97 % of cases, higher than in HIC studies at 47 % to 77 % (Vanvelk et al., 2023; Onsea et al., 2022; Buijs et al., 2022). Postoperative fever is often misattributed to malaria or urinary/respiratory infections, leading to antimalarial and empirical antibiotic use that can mask early-onset FRI (Fonkoué et al., 2024b). Other contributing factors may include patients' low awareness of early signs of FRI, systematic use of antibiotics via self-medication or empirical prescriptions, and delayed presentation. When clinical confirmatory signs do emerge, the initial response is frequently the prescription of antibiotics. Indeed, over 80 % of patients were already on antibiotics before admission, far higher than the 15.1 % reported by Onsea et al. (2022), highlighting the need for improved antibiotic stewardship (Onsea et al., 2022).

Microbiological confirmatory criteria were met in only 21.3 % of patients, and histological criteria were met in 1.2 % of patients (Metsemakers et al., 2018), although analysis was performed in 77.5 % of patients, with 80.2 % positivity. Recommended standards – 5 intraoperative deep tissue samples – were met in 0 % for quantity and 46.6 % for sampling method. With an average of 1.4 samples per patient (range: 1–3), we achieved a positivity rate of 80.2 %. In resource-limited settings where costs are borne by patients, 3 samples may be a practical compromise, yielding 85 % sensitivity versus 97 % for 5 samples (Dudareva et al., 2021), though some pathogens may be missed, particularly in polymicrobial infections (Corrigan et al., 2022). Histopathology may be justified only when clinical confirmation is lacking.

Efforts should focus on raising awareness and early suspicion of FRI. Local studies are needed to define algorithms based on suggestive clinical features and accessible investigations such as WBC, CRP, ESR, plain radiographs, and CT scans. Advanced imaging modalities, such as WBC scintigraphy and ^18^FDG-PET, which are not yet available, could help identify FRIs that may be missed due to the absence of confirmatory criteria; however, it should be noted that these are not mandatory in the FRI consensus definition. Both patients and healthcare providers must be educated to consider FRI in any delayed bone healing, especially after open fractures or prior surgery, regardless of timing.

### 4.2 Temporal classification of FRI

An important context-specific finding is that one-third of FRIs occurred in patients without prior surgery. This scenario is not addressed in the Willenegger and Roth classification (Willenegger and Roth, 1986) or current consensus guidelines (Metsemakers et al., 2018, 2020; Govaert et al., 2020; Marais et al., 2024a). The division of FRIs by time from injury is also controversial. McNally et al. (2022) found no correlation between time from injury and treatment outcome (McNally et al., 2022), and the new consensus FRI classification does not include time from injury (Alt et al., 2024). Likewise, classic strategies like DAIR and DAIEX are not literally applicable without implants; in such cases, we propose an adapted approach: DAIF (debridement, antibiotics, and initial fixation).

### 4.3 Management of FRI

A key observation was the absence of MDTs, despite most centers having necessary specialties except plastic surgery. Multidisciplinary care is well documented to improve outcomes (Rupp et al., 2023; Muller et al., 2022) without significant extra resources.

In two-thirds of cases, FRI treatment deviated from guidelines. A major deviation was the high rate (44.4 %) of suppressive antibiotics without surgery, likely reflecting limited surgical access, physician preference, limited education, and financial constraints. Among surgical treatments, DAIEX (23 %) was more common than DAIR (9.5 %), possibly due to delays in intervention. Surgical treatment was used in only 30 % of early infections, while 70 % of early infections were managed solely with antibiotics and dressings. This may be explained by the inherent challenge for surgeons in recognizing and acknowledging postoperative infectious complications and by the perception that an early return to the operating room represents a therapeutic failure. However, delayed DAIR can compromise implant salvage (Marais et al., 2024a; Baertl et al., 2024; Morgenstern et al., 2021; Vicenti et al., 2024) – a major concern in LMICs where implants are costly. Conversely, a South African study showed that in selected cases of FRI after intramedullary nailing, suppressive antibiotics alone achieved 95 % bone union and 98 % infection remission, despite DAIEX being indicated in these cases in HICs (Tsang et al., 2024). These findings suggest that in resource-limited settings, with stable constructs and adequate soft tissue coverage, not every FRI may require DAIR or DAIEX. Locally driven research is needed to clarify the role of non-surgical strategies in specific scenarios.

Achieving and maintaining adequate mechanical stability is crucial in managing fracture-related infection, as it promotes bone healing, reduces strain at the infection site, and enhances surgical debridement and systemic antibiotic efficacy (Foster et al., 2021). In this study, fixation relied mainly on external fixators, even during second-stage DAIEX procedures. Internal fixation was underutilized, likely due to concerns about reinfection and reoperation burden. This reluctance – aimed at minimizing risk – may compromise construct stability, which is a known factor in infection control. A paradigm shift is needed to encourage the safe use of internal fixation when indicated.

Long-acting local antibiotic carriers that allow single-stage treatment and safer internal fixation could be highly beneficial (Sliepen et al., 2022; McNally et al., 2022) but remain largely unavailable or unaffordable. Training in orthoplastic techniques is also critical for managing soft tissue defects and for improving FRI prevention – especially in open fractures – and treatment outcomes (Fonkoue et al., 2023a; Marais et al., 2024b; McNally et al., 2022).

### 4.4 Limitations

This study has limitations, including a relatively small sample, limited follow-up, and absence of outcome data. However, its primary aim was to evaluate the feasibility of applying consensus recommendations in a resource-limited setting rather than treatment outcomes. The prospective, multicenter design and the unique data provided enhance its relevance for informing future strategies and designing intervention trials.

## 5 Conclusions

This study found that the diagnosis of FRIs in low-resource settings is primarily based on clinical confirmatory criteria. As a result, the number of cases is likely underestimated. The recommendations regarding microbiological analysis – particularly the number and type of samples – appear difficult to implement in this context. Histopathological analysis, although accessible, is not yet routinely included in the diagnostic work-up for these patients. The FRI consensus definition is applicable in LMICs, particularly as it does not exclude patients without prior fracture fixation. Conventional surgical approaches (DAIR and DAIEX) are not applicable for one-third of patients who present with infections without having undergone prior surgery and thus without implant-related involvement. Therefore, DAIF may represent a relevant concept for LMIC-specific scenarios. Two-thirds of FRIs are managed outside international guidelines. This study provides key insights into the factors that could be targeted to improve the diagnosis and management of fracture-related infections in resource-limited settings. Further studies are warranted to identify outcome predictors in this specific context and to develop evidence-based, context-specific algorithms better suited to resource-constrained environments.

## Data Availability

Raw data are available from the corresponding author upon reasonable request.
